# Loss of caveolin-3-dependent regulation of *I*_Ca_ in rat ventricular myocytes in heart failure

**DOI:** 10.1152/ajpheart.00458.2017

**Published:** 2017-11-03

**Authors:** Simon M. Bryant, Cherrie H. T. Kong, Mark B. Cannell, Clive H. Orchard, Andrew F. James

**Affiliations:** School of Physiology, Pharmacology and Neuroscience, University of Bristol, Bristol, United Kingdom

**Keywords:** caveolin-3, cAMP, heart failure, L-type Ca^2+^ current, myocardial infarction

## Abstract

β_2_-Adrenoceptors and L-type Ca^2+^ current (*I*_Ca_) redistribute from the t-tubules to the surface membrane of ventricular myocytes from failing hearts. The present study investigated the role of changes in caveolin-3 and PKA signaling, both of which have previously been implicated in this redistribution. *I*_Ca_ was recorded using the whole cell patch-clamp technique from ventricular myocytes isolated from the hearts of rats that had undergone either coronary artery ligation (CAL) or equivalent sham operation 18 wk earlier. *I*_Ca_ distribution between the surface and t-tubule membranes was determined using formamide-induced detubulation (DT). In sham myocytes, β_2_-adrenoceptor stimulation increased *I*_Ca_ in intact but not DT myocytes; however, forskolin (to increase cAMP directly) and H-89 (to inhibit PKA) increased and decreased, respectively, *I*_Ca_ at both the surface and t-tubule membranes. C3SD peptide (which decreases binding to caveolin-3) inhibited *I*_Ca_ in intact but not DT myocytes but had no effect in the presence of H-89. In contrast, in CAL myocytes, β_2_-adrenoceptor stimulation increased *I*_Ca_ in both intact and DT myocytes, but C3SD had no effect on *I*_Ca_; forskolin and H-89 had similar effects as in sham myocytes. These data show the redistribution of β_2_*-*adrenoceptor activity and *I*_Ca_ in CAL myocytes and suggest constitutive stimulation of *I*_Ca_ by PKA in sham myocytes via concurrent caveolin-3-dependent (at the t-tubules) and caveolin-3-independent mechanisms, with the former being lost in CAL myocytes.

**NEW & NOTEWORTHY** In ventricular myocytes from normal hearts, regulation of the L-type Ca^2+^ current by β_2_-adrenoceptors and the constitutive regulation by caveolin-3 is localized to the t-tubules. In heart failure, the regulation of L-type Ca^2+^ current by β_2_-adrenoceptors is redistributed to the surface membrane, and the constitutive regulation by caveolin-3 is lost.

## INTRODUCTION

L-type Ca^2+^ current (*I*_Ca_) plays a key role in excitation-contraction (EC) coupling in cardiac ventricular myocytes: activation of L-type Ca^2+^ channels (LTCCs) during the action potential causes influx of Ca^2+^ that triggers Ca^2+^ release via ryanodine receptors (RyRs) in the adjacent sarcoplasmic reticulum (SR) membrane (2, 8). Previous work has shown that the function of many of the key proteins involved in EC coupling, including LTCCs and RyRs, occurs predominantly at the t-tubules: invaginations of the surface membrane that enable near-synchronous SR Ca^2+^ release, and thus contraction, throughout the cell (18, 21, 28). The mechanism for the localization of *I*_Ca_ at the t-tubules is less clear, although it has been suggested that the caveolar protein caveolin-3 (Cav-3) plays a role in the localization of *I*_Ca_, possibly via a mechanism involving cAMP/PKA signaling pathways (1, 5, 9, 24).

Cav-3 is also involved in the localization of cAMP signaling via β_2_-adrenoceptors to the t-tubules, and it has been proposed that LTCCs and β_2_-adrenoceptors are colocalized in a Cav-3 signaling microdomain (1, 5, 7, 23, 30). It has been shown that Cav-3 plays a critical role in the constitutive maintenance of *I*_Ca_ at the t-tubule (5). In heart failure, there is redistribution of β_2_-adrenoceptors from the t-tubular to the surface membrane so that they become more uniformly distributed across the cell membrane (22, 27). This redistribution is associated with a change from localized to more diffuse signaling in response to β_2_-adrenergic stimulation (27). We (6) have recently shown in a coronary artery ligation (CAL) model in that rat that ventricular *I*_Ca_ is also redistributed from the t-tubules to the surface sarcolemma in heart failure.

We hypothesized that the redistribution of *I*_Ca_ after CAL is due to loss of Cav-3-dependent localization at the t-tubules, which may be secondary to the decreased expression of Cav-3 observed in heart failure. Thus, changes in the localization of the β_2_-signaling pathway in heart failure may be associated with a loss of constitutive regulation of *I*_Ca_ by PKA at the t-tubules. Therefore, we further investigated the relationship between the distribution of *I*_Ca_ and changes in Cav-3/β_2_-adrenergic signaling observed after CAL in rats (6).

## METHODS

### 

#### Animals and surgical procedures.

All procedures were performed in accordance with United Kingdom legislation and approved by the University of Bristol Ethics Committee. This study was conducted in parallel with other investigations using cells from the same animals to investigate ventricular and atrial cellular remodeling in heart failure and thereby conformed with the reduction component of the 3Rs (“replace, reduce, refine”) (3, 6, 16). Adult male Wistar rats (~250 g) were subjected to either ligation of the left anterior descending coronary artery (CAL; 10 animals) or equivalent surgery without ligation (sham; 12 animals). Operations were conducted under general anesthesia [ketamine (75 mg/kg) and medetomidine (0.5 mg/kg ip)] with appropriate analgesia [buprenorphine (0.05 mg/kg sc)], as previously described (6). Data regarding changes in cardiac morphology and function as well as in cell morphology in these groups of animals have been previously published (3, 6).

#### Myocyte isolation.

Left ventricular myocytes were isolated from the hearts ~18 wk after surgery as previously described (5). Animals were euthanized under pentobarbitone anesthesia, and the heart was quickly excised and Langendorff perfused at 8 ml/min (37°C), initially with Tyrode solution (see *Solutions* below) plus 0.75 mmol/l CaCl_2_ for 4 min and then in nominally Ca^2+^-free solution for 4 min and finally plus 1 mg/ml collagenase (Worthington) for 10 min. The left ventricle was then excised and shaken in collagenase-containing solution at 37°C for 5–7 min, filtered, and centrifuged. The supernatant was discarded, and the pellet was resuspended in Kraftbrühe solution and stored at 4°C for 2–10 h before use on the day of isolation (20). Detubulation (DT) of myocytes (physical and functional uncoupling of the t-tubules from the surface membrane) was achieved using formamide-induced osmotic shock, as previously described (21).

#### Solutions.

Tyrode solution for cell isolation contained (in mmol/l) 130 NaCl, 5.4 KCl, 0.4 NaH_2_PO_4_, 4.2 HEPES, 10 glucose, 1.4 MgCl_2_, 20 taurine, and 10 creatinine; pH 7.4 (NaOH). Kraftbrühe solution for cell storage contained (in mmol/l) 90 l-glutamic acid, 30 KCl, 10 HEPES, 1 EGTA, 5 Na pyruvate, 20 taurine, 20 glucose, 5 MgCl_2_, 5 succinic acid, 5 creatine, 2 Na_2_ATP, and 5 β-OH butyric acid; pH 7.4 with KOH. For patch-clamp experiments, cells were superfused with solution that contained (in mmol/l) 133 NaCl, 1 MgSO_4_, 1 CaCl_2_, 1 Na_2_HPO_4_, 10 glucose, 10 HEPES [pH 7.4 (NaOH)]; 5 mmol/l CsCl was added to inhibit K^+^ currents. The pipette solution contained (in mmol/l) 110 CsCl, 20 TEA-Cl, 0.5 MgCl_2_, 5 Mg-ATP, 5 BAPTA, 10 HEPES, and 0.4 GTP-Tris; pH 7.2 (CsOH). BAPTA was used to inhibit Ca^2+^-dependent inactivation of *I*_Ca_ (33).

Selective β_2_-adrenoceptor stimulation was achieved as previously described (5) using the β_2_-adrenoceptor agonist zinterol (1 and 3 μmol/l) in the presence of the β_1_-adrenoceptor-selective antagonist atenolol (10 μmol/l); cells were superfused with atenolol alone for at least 4 min before superfusion with zinterol in the presence of atenolol. Under these conditions, the effects of 1 and 3 μmol/l zinterol could be completely abolished by 100 nM ICI-118,551, a β_2_-adrenoceptor-selective antagonist (5). The plant alkaloid forskolin (10 μmol/l) was used to activate adenylyl cyclase directly (31). C3SD, a short peptide encompassing the Cav-3 scaffolding domain, was used to disrupt binding of Cav-3 to its protein partners as previously described (5, 13, 15, 23); myocytes were incubated in 1 µmol/l TAT-C3SD for at least 45 min before use. PKA was inhibited using H-89 (20 μmol/l) (11, 17).

#### Recording and analysis of I_Ca_.

Myocytes were placed in a chamber mounted on a Nikon Diaphot inverted microscope. Membrane currents and cell capacitance were recorded using the whole cell patch-clamp technique using an Axopatch 200B, Digidata 1322A analog-to-digital converter, and pClamp 10 (Axon Instruments). Pipette resistance was typically 2–4 MΩ when filled with pipette solution, and pipette capacitance and series resistance were compensated by ~70%. Currents were activated from a holding potential of −80 mV by a 100-ms step depolarization to –40 mV (to inactivate Na^+^ current) followed by steps to potentials between −50 and +80 mV for 500 ms before repolarization to the holding potential at a frequency of 0.2 Hz. *I*_Ca_ amplitude (in pA) was measured as the difference between peak inward current and current at the end of the depolarizing pulse and was normalized to cell capacitance [in pF; a function of membrane area (25)] to calculate *I*_Ca_ density (in pA/pF). Surface membrane current density was obtained from currents measured in DT myocytes, whereas t-tubular membrane current density was calculated by subtraction of surface from whole cell currents and corrected for incomplete DT, as previously described (5, 6, 19, 21). DT efficiency, measured from images of intact and DT cells stained with di-8-ANEPPS, was ~84% and was not different between wild-type and CAL myocytes (6). To correct for incomplete DT, the distribution of membrane capacitance and *I*_Ca_ between the t-tubule and surface membrane was calculated as previously described (6). As we have previously reported, there was no statistically significant difference between sham and CAL myocytes in the degree of osmotic shock-induced DT, nor was there any relationship between the whole cell capacitance and time of recording (6).

#### Statistics.

Data are expressed as means ± SE of *n* myocytes. Statistical analysis was performed using GraphPad Prism (GraphPad Software). *I*_Ca_ density-voltage relationship curves were analyzed using repeated-measures ANOVA with voltage and the corresponding intervention (i.e., DT, H-89, or C3SD) as factors. *I*_Ca_ properties elicited by a step depolarization to a single voltage were analyzed by two-way ANOVA. Post hoc tests used the Bonferroni correction. The limit of statistical confidence was taken as *P* < 0.05. Errors in derived variables (specifically *I*_Ca_ density at the t-tubule membrane) and the subsequent statistical analysis (unpaired Student’s *t*-test) were calculated using propagation of errors from the source measurements (6, 14).

## RESULTS

### 

#### Effect of CAL on the response to β_2_-adrenoceptor stimulation.

**Fig. 1.**
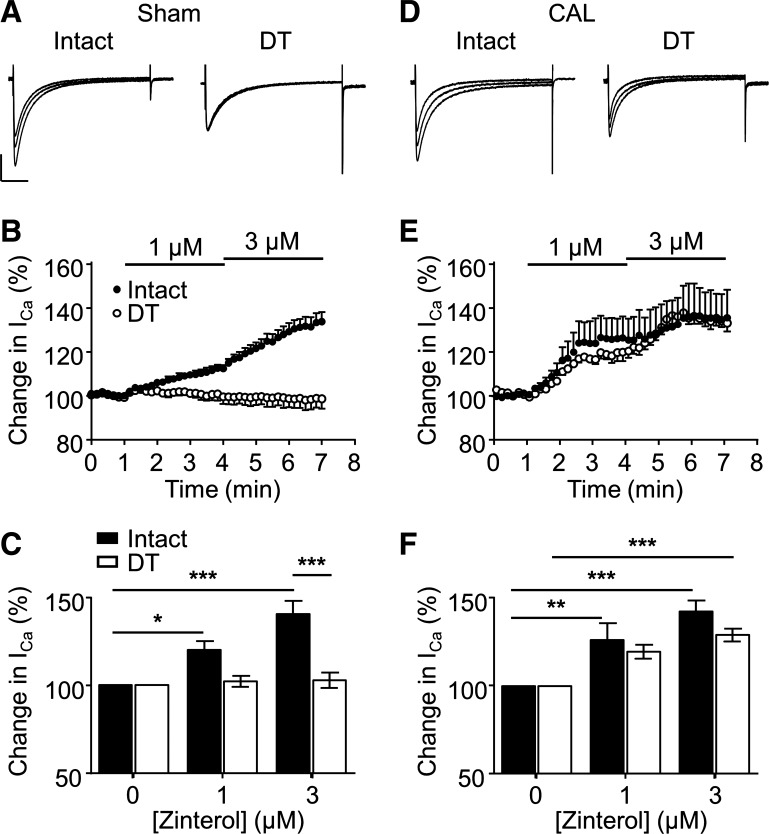
β_2_-Adrenergic potentiation of L-type Ca^2+^ current (*I*_Ca_) in sham and coronary artery ligated (CAL) myocytes. *A*: representative *I*_Ca_ traces (elicited by step depolarization to 0 mV) recorded from intact and detubulated (DT) myocytes isolated from sham hearts. Overlapping traces were from the same cell and were recorded under control conditions and after application of 1 and 3 µmol/l zinterol (in the presence of 10 µmol/l atenolol). Vertical scale bar = 1 nA; horizontal scale bar = 50 ms. *B*: time course of changes in mean normalized peak *I*_Ca_ (±SE) of intact (*n* = 5) and DT (*n* = 6) sham myocytes during superfusion with control solution (containing 10 μmol/l atenolol) and 1 and 3 µmol/l zinterol. *I*_Ca_, elicited by step depolarization to 0 mV at 0.1 Hz, was expressed as a percentage of control measured just before application of the first concentration of zinterol. *C*: mean changes in *I*_Ca_ elicited by application of 1 and 3 µmol/l zinterol to intact (1 μmol/l: *n* = 7 and 3 μmol/l: *n* = 9) and DT (1 μmol/l: *n* = 7 and 3 μmol/l: *n* = 9] sham myocytes. Data were subjected to two-way ANOVA: β_2_-agonism *P* < 0.001; DT *P* < 0.001; interaction *P* < 0.001. *P < 0.05 and ***P < 0.001, Bonferroni post hoc test. *D*: representative *I*_Ca_ traces (elicited by step depolarization to 0 mV) recorded from intact and DT myocytes isolated from CAL hearts. Conditions and scale were as in *A*. *E*: time course of changes in mean normalized peak *I*_Ca_ of intact (*n* = 5) and DT (*n* = 4) CAL myocytes during superfusion with control solution (containing 10 μmol/l atenolol) and 1 and 3 μmol/l zinterol. *F*: mean changes in *I*_Ca_ elicited by application of 1 and 3 µmol/l zinterol to intact (1 μmol/l: *n* = 5 and 3 μmol/l: *n* = 19) and DT (1 μmol/l: *n* = 4 and 3 μmol/l: *n* = 8) CAL myocytes. Data were subjected to two-way ANOVA: β_2_-agonism *P* < 0.001, DT not significant, interaction not significant. **P < 0.01 and ***P < 0.001, Bonferroni post hoc test.

In intact ventricular myocytes from sham hearts, selective activation of β_2_-adrenoceptors (1 and 3 μmol/l zinterol in the presence of 10 μmol/l atenolol) caused a significant, concentration-dependent increase of *I*_Ca_, which reached ~140% of control in the steady state in the presence of 3 μmol/l of the β_2_-agonist (Fig. 1*A*, *left*, *B*, and *C*). In contrast, in DT cells, 3 μmol/l zinterol did not increase *I*_Ca_ (Fig. 1*A*, *right*, *B*, and *C*). In CAL myocytes, 3 μmol/l zinterol caused an increase of ~40% in intact myocytes and ~29% in DT myocytes (Fig. 1, *D–F*). Thus, because *I*_Ca_ recorded in DT cells represents the current at the surface membrane, it appears that in sham myocytes, the response of *I*_Ca_ to β_2_-adrenoceptor stimulation occurs predominantly at the t-tubule membrane. However, after CAL, the β_2_-adrenergic response redistributes and occurs at both the cell surface and t-tubule membranes. These data also show that the DT procedure per se is not responsible for the lack of response to zinterol observed in sham myocytes.

#### Effect of CAL on the response to forskolin.

**Fig. 2.**
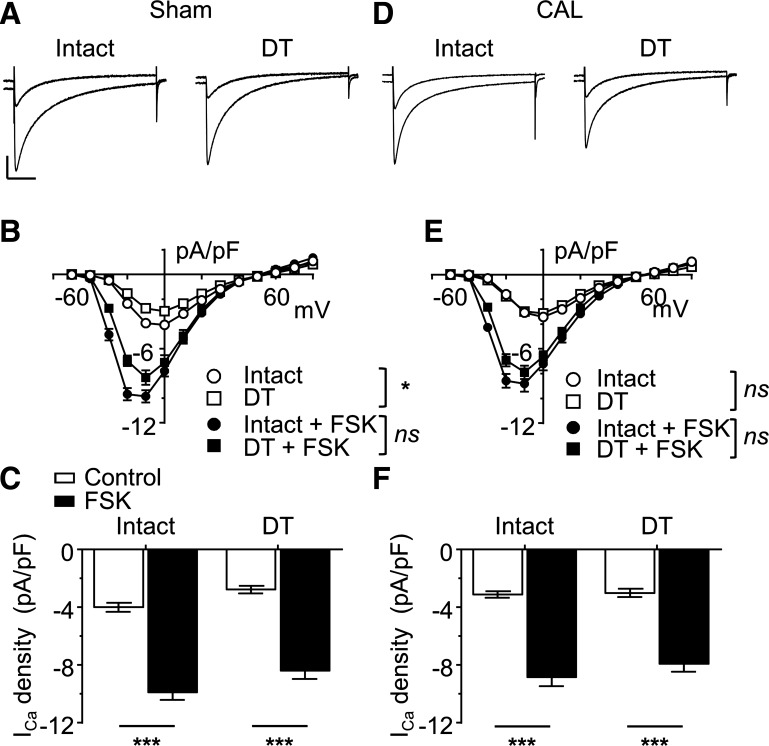
Increase in L-type Ca^2+^ current (*I*_Ca_) through direction activation of adenylyl cyclase in sham and coronary artery ligated (CAL) myocytes. *A*: representative *I*_Ca_ traces (elicited by step depolarization to 0 mV) recorded in the absence and presence of forskolin (FSK; 10 μmol/l and 0.5 mmol/l CaCl_2_) from intact and detubulated (DT) myocytes isolated from sham hearts. Overlapping traces were taken from the same myocytes under control conditions and after 3-min perfusion with FSK. Vertical scale bar = 2 pA/pF; horizontal scale bar = 100 ms. *B*: mean *I*_Ca_ density-voltage relationships from intact (*n* = 12) and DT (*n* = 14) sham myocytes in the absence and presence of FSK. Not significant (ns), *P >* 0.05, *P < 0.05; two-way ANOVA with Bonferroni post hoc test, intact vs. DT cells. *C*: effect of FSK on peak *I*_Ca_ density (elicited at −10 mV) recorded in intact and DT sham myocytes. Data were subjected to two-way ANOVA: FSK *P* < 0.001, DT *P* < 0.01, interaction ns. ***P < 0.001, Bonferroni post hoc test. *D*: representative *I*_Ca_ traces recorded in the absence and presence of FSK (10 μmol/l and 0.5 mmol/l CaCl_2_) from intact and DT myocytes isolated from CAL hearts. Conditions and scale were as in *A*. *E*: mean *I*_Ca_ density-voltage relationships from intact (*n* = 18) and DT (*n* = 12) CAL myocytes in the absence and presence of FSK. ns, *P >* 0.05, *two-way ANOVA with Bonferroni post hoc test, intact vs. DT cells. *F*: effect of FSK on peak *I*_Ca_ density (elicited at −10 mV) recorded in intact and DT CAL myocytes. Data were subjected to two-way ANOVA: FSK *P* < 0.001, DT *P* < 0.01, interaction ns. ***P < 0.001, Bonferroni post hoc test.

To investigate whether the distribution of the β_2_-adrenergic response was due to localization of a downstream component of the signaling pathway, we used forskolin (10 μmol/l) to activate adenylyl cyclase directly, to increase cAMP in the absence of adrenoceptor stimulation. Superfusion with forskolin (10 μmol/l) increased *I*_Ca_ in both intact and DT myocytes from sham hearts (Fig. 2*A*). The corresponding mean *I*_Ca_ density-voltage relationships for intact and DT myocytes are shown in Fig. 2*B*. Figure 2*C* shows the effect of forskolin on *I*_Ca_ density at a test potential of −10 mV in sham intact and DT myocytes. Forskolin also caused an increase in *I*_Ca_ in both intact and DT myocytes from CAL hearts (Fig. 2, *D–F*). These data show that the increase in *I*_Ca_ in response to forskolin was similar in intact sham and CAL myocytes. More importantly, these data also show that forskolin caused a significant increase in the amplitude of *I*_Ca_ in DT sham myocytes, which was similar to that observed in DT CAL myocytes. Thus, it appears that adenylyl cyclase and PKA are present at both the surface and t-tubule membranes in both sham and CAL myocytes and can stimulate *I*_Ca_ to a similar extent at either site. It is unlikely, therefore, that the lack of effect of zinterol in DT sham myocytes was due to absence of the components of the cAMP signaling pathway (i.e., adenylyl cyclase and PKA) at the cell surface but may be due to the absence of β_2_-adrenoreceptors.

#### Effect of CAL on the response to C3SD.

**Fig. 3.**
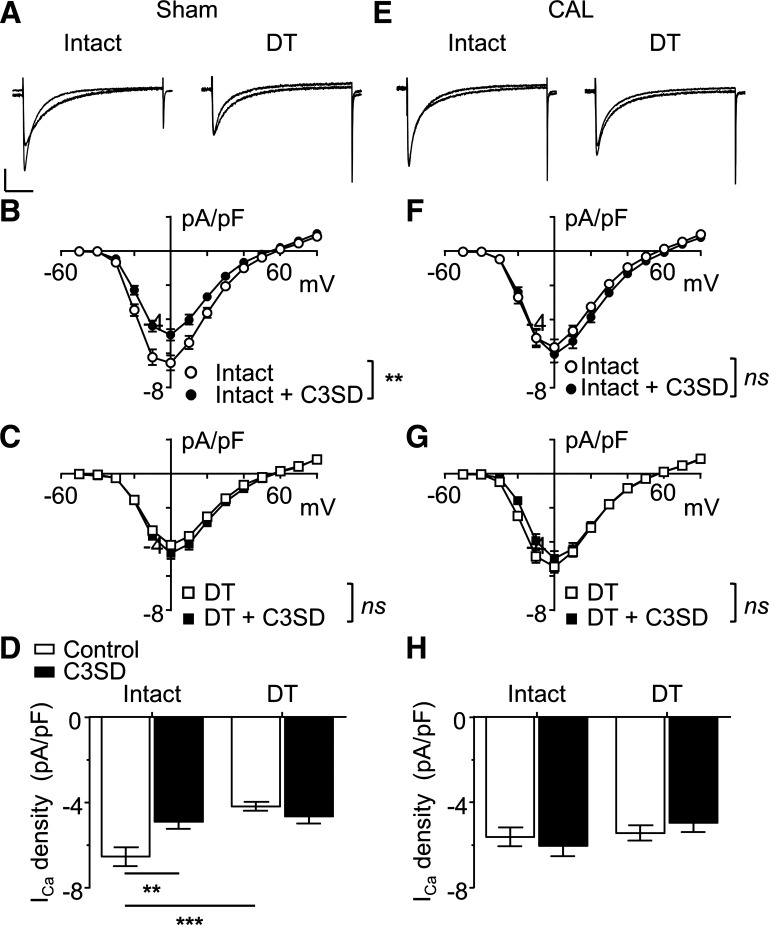
Constitutive regulation of basal L-type Ca^2+^ current (*I*_Ca_) by caveolin-3 (Cav-3). *A*: representative *I*_Ca_ traces recorded from intact and detubulated (DT) myocytes isolated from sham hearts. Overlapping traces were taken from different myocytes that had either undergone incubation with C3SD peptide (1 µmol/l) or were untreated. Vertical scale bar = 2 pA/pF; horizontal scale bar = 100 ms. *B*: mean *I*_Ca_ density-voltage relations from untreated intact sham cells (*n* = 16) and intact sham cells treated with C3SD peptide (*n* = 16). **P < 0.01, two-way ANOVA with Bonferroni post hoc test, untreated vs. C3SD-treated cells. *C*: mean *I*_Ca_ density-voltage relations from untreated sham DT cells (*n* = 20) and sham DT cells treated with C3SD peptide (*n* = 10). Not significant (ns), *P >* 0.05; two-way ANOVA with Bonferroni post hoc test, untreated vs. C3SD-treated cells. *D*: effect of C3SD on peak *I*_Ca_ density (elicited at 0 mV) recorded from intact and DT sham myocytes. Data were subject to two-way ANOVA: C3SD ns, DT *P* < 0.001, interaction *P* < 0.01. **P < 0.01 and ***P < 0.001, Bonferroni post hoc test. *E*: representative *I*_Ca_ traces recorded from intact and DT myocytes isolated from coronary artery ligated (CAL) hearts. Conditions and scale were as in *A*; overlapping traces were taken from different myocytes that had either undergone incubation with C3SD peptide (1 µmol/l) or were untreated. *F*: mean *I*_Ca_ density-voltage relations from untreated intact CAL cells (*n* = 14) and intact CAL cells treated with C3SD peptide (*n* = 15). ns, *P >* 0.05, two-way ANOVA with Bonferroni post hoc test, untreated vs. C3SD-treated cells. *G*: mean *I*_Ca_ density-voltage relations from untreated DT CAL cells (*n* = 22) and DT CAL cells treated with C3SD peptide (*n* = 7). ns, *P >* 0.05, two-way ANOVA with Bonferroni post hoc test, untreated vs. C3SD-treated cells. *H*: effect of C3SD on peak *I*_Ca_ density (elicited at 0 mV) recorded from intact and DT CAL myocytes. Data were subjected to two-way ANOVA: C3SD ns, DT ns, interaction ns.

Since Cav-3 has been implicated in the localization of β_2_-adrenoceptor/cAMP signaling at the t-tubules, we investigated the effect of acutely inhibiting Cav-3 binding to its partner proteins by pretreatment of cells with C3SD peptide (5, 15). *I*_Ca_ density was reduced in intact sham myocytes treated with the C3SD peptide (Fig. 3*A*, *left*, and *B*). However, treatment with C3SD had no effect on *I*_Ca_ density in DT sham myocytes (Fig. 3*A*, *right*, and *C*). The effects of treatment with C3SD on *I*_Ca_ density at 0 mV in intact and DT myocytes from sham hearts are shown in Fig. 3*D*. In contrast to its effect in sham myocytes, C3SD had no effect on *I*_Ca_ density in intact CAL myocytes (Fig. 3*E*, *left*, *F*, and *H*), nor did C3SD have any effect on *I*_Ca_ in DT CAL myocytes (Fig. 3*E*, *right*, *G*, and *H*). Thus, there appears to be no Cav-3-dependent regulation of *I*_Ca_ at the surface membrane in either sham or CAL myocytes. However, there does appear to be Cav-3-dependent stimulation of *I*_Ca_ at the t-tubules of sham myocytes, which is absent in CAL cells.

#### Effect of CAL on the response to H-89 in the absence and presence of C3SD.

**Fig. 4.**
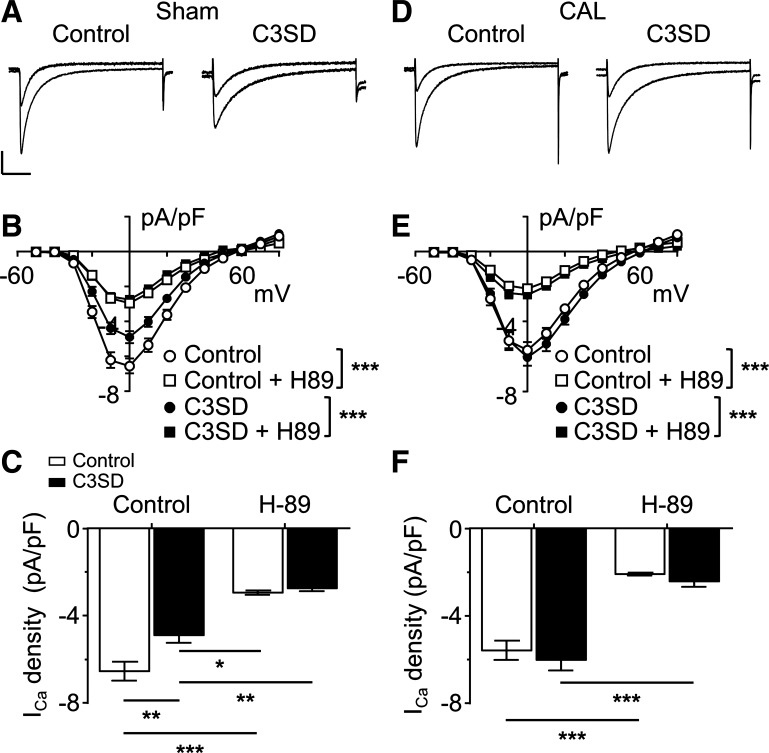
Role of PKA in caveolin-3 (Cav-3)-dependent regulation of basal L-type Ca^2+^ current (*I*_Ca_) in sham and coronary artery ligated (CAL) myocytes. *A*: representative *I*_Ca_ traces recorded from intact untreated (control) and C3SD-treated (C3SD) myocytes isolated from sham hearts. Overlapping traces were taken from the same myocytes before and after application of the PKA inhibitor H-89 (20 µmol/l). Vertical scale bar = 2 pA/pF; horizontal scale bar = 100 ms. *B*: mean *I*_Ca_ density-voltage relationship curves recorded from intact myocytes isolated from sham hearts that were either untreated (*n* = 16) or treated with C3SD (*n* = 16) before and after application of H-89. Control data are the same as those shown in Fig. 3*B*. ***P < 0.001, two-way ANOVA with Bonferroni post hoc test, absence vs. presence of H-89. *C*: effect of PKA inhibition on mean peak *I*_Ca_ density (elicited at 0 mV) in sham myocytes that were untreated or treated with C3SD. Data were subjected to two-way ANOVA: C3SD not significant (ns), H-89 *P* < 0.001, interaction ns. *P < 0.05, **P < 0.01, and ***P < 0.001, Bonferroni post hoc test. *D*: representative *I*_Ca_ traces recorded from untreated and C3SD-treated myocytes isolated from CAL hearts before and after application of the PKA inhibitor H-89 (20 µmol/l). Conditions and scale were as in *A*. *E*: mean *I*_Ca_ density-voltage relationship curves recorded from intact myocytes isolated from CAL hearts that were either untreated (*n* = 14) or treated with C3SD (*n* = 15) before and after application of H-89. Control data are the same as those shown in Fig. 3*F*. ***P < 0.001, two-way ANOVA with Bonferroni post hoc test, absence vs. presence of H-89. *F*: effect of PKA inhibition on mean peak *I*_Ca_ density (elicited at 0 mV) in CAL myocytes that were treated or untreated with C3SD. Data were subjected to two-way ANOVA: C3SD ns, H-89 *P* < 0.001, interaction ns. ***P < 0.001, Bonferroni post hoc test.

Since it has been suggested that Cav-3-dependent stimulation of t-tubular *I*_Ca_ is via a PKA-dependent mechanism (5), we investigated the effect of the PKA inhibitor H-89 on *I*_Ca_ density after CAL and the effect of C3SD on the response to H-89. Inhibition of PKA (20 μmol/l H-89) decreased *I*_Ca_ in untreated and C3SD-treated sham myocytes, indicating that constitutive stimulation of *I*_Ca_ by PKA that did not require Cav-3 (Fig. 4, *A*–*C*). Moreover, there was no difference in the *I*_Ca_ density-voltage relations of untreated and C3SD-treated cells in the presence of H-89 (Fig. 4*B*), demonstrating that the effects of H-89 and C3SD treatment were not summative. Thus, in the presence of PKA inhibition, treatment with C3SD peptide was without effect on *I*_Ca_ density, indicating that PKA activity was required for the constitutive regulation of *I*_Ca_ by Cav-3 in sham myocytes. Similarly, H-89 decreased *I*_Ca_ density to the same level in both untreated and C3SD-treated CAL myocytes, indicating constitutive regulation of *I*_Ca_ by PKA (Fig. 4, *D*–*F*). C3SD was without effect on *I*_Ca_ density in either the absence or presence of H-89 (Fig. 4, *E* and *F*). These data show that in sham myocytes, there is constitutive stimulation of *I*_Ca_ by PKA that is mediated both via Cav-3-dependent (localized to the t-tubule membrane) and Cav-3-independent mechanisms. Although the constitutive regulation by Cav-3 was lost in CAL myocytes, constitutive regulation of *I*_Ca_ via PKA remained.

#### Effect of DT on the constitutive regulation of I_Ca_ by PKA.

**Fig. 5.**
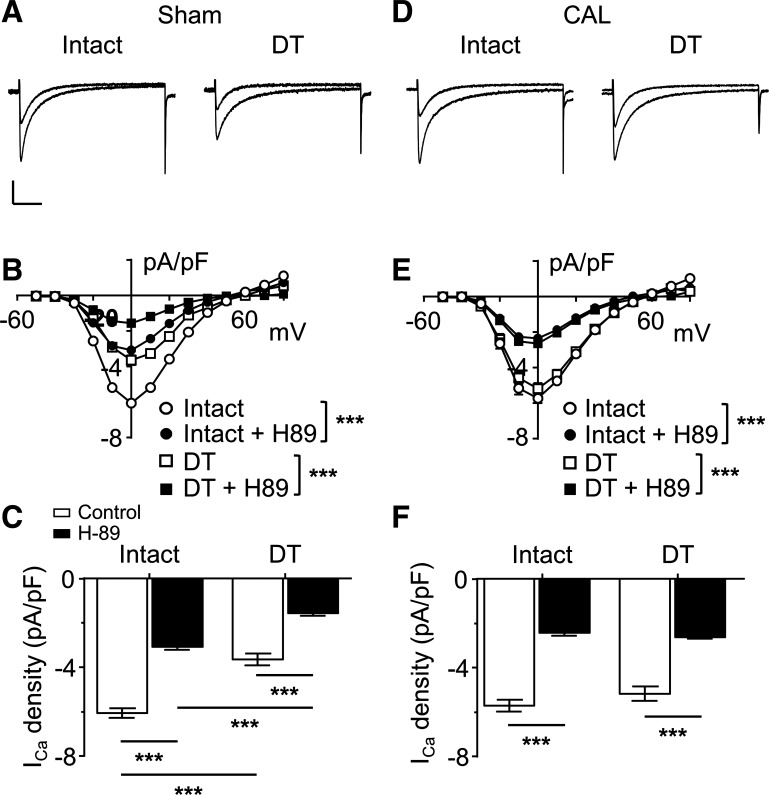
Localization of PKA-dependent regulation of basal L-type Ca^2+^ current (*I*_Ca_) in sham and coronary artery ligated (CAL) myocytes. *A*: representative *I*_Ca_ traces recorded from intact and detubulated (DT) myocytes isolated from sham hearts. Overlapping traces were taken from the same myocytes before and after application of the PKA inhibitor H-89 (20 µmol/l). Vertical scale bar = 2 pA/pF; horizontal scale bar = 100 ms. *B*: mean *I*_Ca_ density-voltage relationship curves recorded from myocytes isolated from sham hearts that were either intact (*n* = 17) or DT (*n* = 8) before and after application of H-89. ***P < 0.001, two-way ANOVA with Bonferroni post hoc test, control vs. H-89. *C*: effect of PKA inhibition on mean peak *I*_Ca_ density (elicited at 0 mV) in intact or DT sham myocytes under control conditions or after PKA inhibition (H-89). Data were subjected to two-way ANOVA: H-89 *P* < 0.001, DT *P* < 0.001, interaction *P* < 0.05. ***P < 0.001, Bonferroni post hoc test. *D*: representative *I*_Ca_ traces recorded from intact and DT myocytes isolated from CAL hearts; overlapping traces were taken from the same myocytes before and after application of the PKA inhibitor H-89. Conditions and scale were as in *A*. *E*: mean *I*_Ca_ density-voltage relationship curves recorded from myocytes isolated from CAL hearts that were either intact (*n* = 14) or DT (*n* = 9) before and after application of H-89. ***P < 0.001, two-way ANOVA with Bonferroni post hoc test, control vs. H-89. *F*: effect of PKA inhibition on mean peak *I*_Ca_ density (elicited at 0 mV) in intact or DT sham myocytes under control conditions or after PKA inhibition (H-89). Data were subjected to two-way ANOVA: H-89 *P* < 0.001, DT not significant, interaction not significant. ***P < 0.001, Bonferroni post hoc test.

To further investigate the site of constitutive PKA-dependent regulation, the response to H-89 was determined in DT myocytes. *I*_Ca_ density was reduced by H-89 in both intact and DT sham myocytes (Fig. 5, *A*–*C*). *I*_Ca_ density was also reduced by DT in both the presence or absence of PKA inhibition (Fig. 5*C*). H-89 also reduced *I*_Ca_ density in intact and DT myocytes from CAL hearts (Fig. 5, *D*–*F*). However, in contrast to sham myocytes, in CAL, *I*_Ca_ density was similar in intact and DT myocytes in either the presence or absence of PKA inhibition (Fig. 5*F*).

**Fig. 6.**
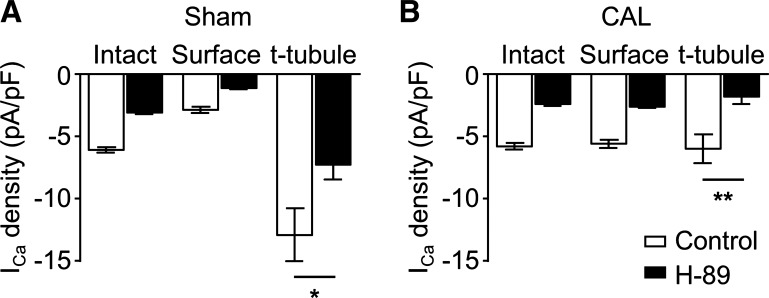
*A*: mean L-type Ca^2+^ current (*I*_Ca_) density at 0 mV measured in intact (total cell membrane) and detubulated (DT) cells (surface membrane) and calculated at the t-tubules (t-tubule membrane) for sham myocytes. Correction for incomplete detubulation was applied (see methods). Control conditions and treatment with H-89 are shown. *B*: mean *I*_Ca_ density at 0 mV measured in intact (total cell membrane) and DT cells (surface membrane) and calculated at the t-tubules (t-tubule membrane) for coronary artery ligated (CAL) myocytes. Correction for incomplete detubulation was applied (see methods). Control conditions and treatment with H-89 are shown. *P < 0.05 and **P < 0.01, Student’s *t*-test.

The calculated current densities at the cell surface and in the t-tubule membrane before and after inhibition of PKA are shown in Fig. 6. These data show that in sham myocytes without inhibition of PKA, *I*_Ca_ density was significantly greater in the t-tubule membrane than at the cell surface, consistent with previous reports (5, 6, 16). In contrast, in CAL myocytes, there was no difference in *I*_Ca_ density between t-tubule and surface membranes (Fig. 6*B*). Inhibition of PKA caused a broadly similar fractional decrease in *I*_Ca_ at the surface membrane in both sham and CAL myocytes, so that surface membrane *I*_Ca_ remained larger in CAL than sham myocytes. Thus, constitutive stimulation of basal *I*_Ca_ by PKA at the cell surface was similar in the two cell types. H-89 also decreased t-tubular *I*_Ca_ density in both cell types so that it was smaller in CAL than sham myocytes. However, after inhibition of PKA, *I*_Ca_ density remained higher at the t-tubules than in the surface membrane in sham myocytes, whereas in CAL myocytes, *I*_Ca_ density was smaller at the t-tubules than at the surface membrane. Thus, it appears not only that *I*_Ca_ redistributes from the t-tubules to the cell surface in heart failure but also that constitutive regulation of t-tubular *I*_Ca_ by PKA is increased in these cells (cf. Fig. 6, *A* and *B*).

## DISCUSSION

**Fig. 7.**
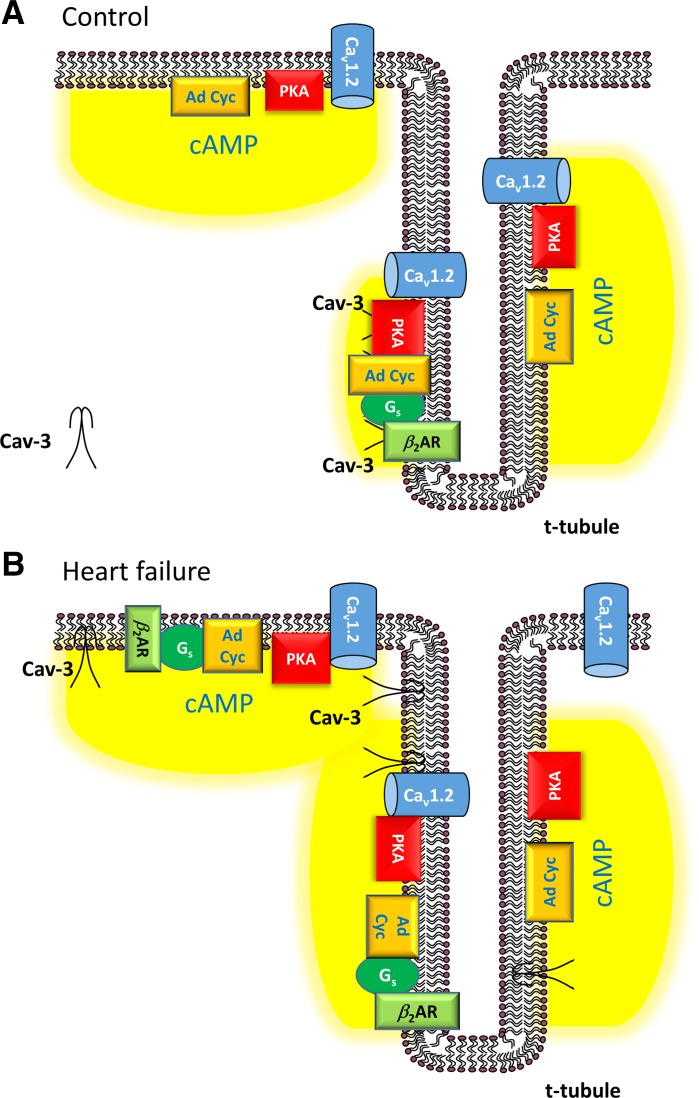
Schema summarizing the role of caveolin-3 (Cav-3) in the regulation of L-type Ca^2+^ current (*I*_Ca_) in normal ventricular myocytes and in heart failure. *A*: regulation of *I*_Ca_ in normal cardiac myocytes. L-type Ca^2+^ channel (LTCC) density is greatest in the t-tubules, where Cav-3 coordinates a signaling domain involving β_2_-adrenoceptors (β_2_ARs), adenylyl cyclase (Ad Cyc), PKA, and the LTCC α_1c_-subunit Ca_v_1.2. β_2_-Adrenoceptors coupled with LTCCs are located exclusively in the t-tubules. Adenylyl cyclase, PKA, and Ca_v_1.2 are also located outside of Cav-3 signaling domains, both within and without t-tubules. Activation of adenylyl cyclase, either via β_2_-adrenoceptors or directly, augments LTCC activity through the production of cAMP. *B*: remodeling of *I*_Ca_ regulation in heart failure. The Cav-3 signaling complex is disrupted. β_2_-Adrenoceptors are located both within the t-tubules and on the surface sarcolemma. LTCC density is more evenly distributed between t-tubules and surface sarcolemma. The role of Cav-3 in the regulation of *I*_Ca_ is lost in heart failure. Schema represents the simplest explanation of the data. Other mechanisms are possible; for example, β_2_-adrenoceptors may be located in both the cell surface and t-tubule membranes in normal cardiac myocytes, but the coupling of β_2_-adrenoceptors with LTCCs is confined to the t-tubules.

This study presents two novel findings regarding the regulation of *I*_Ca_ in heart failure. First, stimulation of *I*_Ca_ by β_2_-adrenoceptors, but not by adenylyl cyclase/PKA, is localized to the t-tubules in sham myocytes and redistributes to the cell surface after CAL. Second, it demonstrates constitutive stimulation of *I*_Ca_ by PKA in sham myocytes that is mediated both via Cav-3-dependent (at the t-tubules) and Cav-3-independent mechanisms, whereas in CAL myocytes, constitutive regulation by Cav-3 is lost, although that via PKA remains at both sites. Thus, the present study advances previous findings from our laboratory that Cav-3 plays a role in the regulation of *I*_Ca_ at the t-tubule by PKA and β_2_-adrenoceptors in normal myocytes (5, 9) and that *I*_Ca_ is redistributed from the t-tubules to the surface sarcolemma in CAL-induced heart failure (6). Interestingly, although constitutive PKA-dependent stimulation of *I*_Ca_ at the cell surface appeared to be the same in both sham and CAL myocytes, constitutive stimulation of t-tubular *I*_Ca_ appeared to increase in CAL myocytes, helping to maintain t-tubular *I*_Ca_. Figure 7 shows schematic diagrams illustrating the regulation of *I*_Ca_ by β_2_-adrenoceptors, Cav-3, and PKA in normal cells (Fig. 7*A*) and in heart failure (Fig. 7*B*).

### 

#### Localization of I_Ca_ regulation by PKA in sham myocytes.

The Ca_v_1.2 pore-forming α-subunit of ventricular LTCCs has been shown to be colocalized with Cav-3, adenylyl cyclase, PKA, and the β_2_-adrenoceptor (1). Stimulation of β_2_-adrenoceptors in cardiac myocytes activates adenylyl cyclase, causing a local increase of cAMP, activation of PKA, and thereby phosphorylation and stimulation of colocalized LTCCs (1). The present data show that β_2_-adrenoceptor stimulation of *I*_Ca_ in sham myocytes occurs predominantly at the t-tubules (Fig. 1), although direct activation of adenylyl cyclase using forskolin increased *I*_Ca_ at both the t-tubular and surface membranes (Fig. 2). Thus, in normal myocytes, adenylyl cyclase and the downstream pathway is present at both the t-tubular and surface membranes, but the β_2_-adrenoceptor is present only at the t-tubules, consistent with previous work showing t-tubular localization of β_2_-adrenoceptor signaling in normal ventricular myocytes (27). Pretreatment with C3SD peptide decreased basal *I*_Ca_ at the t-tubules but not at the surface membrane in sham myocytes (Fig. 3), showing that Cav-3 plays a role in the constitutive stimulation of *I*_Ca_ at the t-tubules but not at the surface membrane in normal cells (Fig. 7*A*). These data are entirely consistent with our previous report (5) in which we showed that pretreatment with C3SD abolished both the constitutive regulation of *I*_Ca_ at the t-tubule and the response to β_2_-adrenoceptors in myocytes from unoperated animals. In contrast, inhibition of PKA using H-89 in the present study decreased *I*_Ca_ at both the surface and t-tubule membranes, presumably reflecting the loss of tonic activity of the adenylyl cyclase/cAMP/PKA pathway at both the surface and t-tubular membranes (Figs. 5 and 7*A*). Although it has been suggested that H-89 may have nonspecific effects independent of PKA inhibition (26), we (9) have previously shown that basal *I*_Ca_ was decreased by a peptide inhibitor of PKA, PKI. Moreover, we have recently shown that H-89 was without effect on basal *I*_Ca_ in rat atrial myocytes from the same hearts as used in the present study, demonstrating both regional differences in the role of PKA in the regulation of *I*_Ca_ and that H-89 was without direct effect on *I*_Ca_ per se (3). The regulation of basal *I*_Ca_ by constitutive PKA activity has also been previously demonstrated in rat ventricular myocytes (4, 5, 9).

While the inhibitory effect of H-89 in sham myocytes was not abolished by pretreatment of the cells with C3SD, H-89 reduced basal *I*_Ca_ to the same mean amplitude in C3SD-treated and untreated cells (Fig. 4), indicating that the effects of C3SD and H-89 were not summative. Thus, PKA is required for the constitutive regulation of *I*_Ca_ by Cav-3 at the t-tubules in sham myocytes, but there is an additional Cav-3-independent constitutive regulation of *I*_Ca_ by PKA. As basal *I*_Ca_ density in DT sham myocytes was reduced by H-89 but not by C3SD, it can be concluded that PKA is also involved in the constitutive regulation of *I*_Ca_ at the surface sarcolemma through a mechanism independent of Cav-3. Taken together, these data suggest a role for Cav-3 in coordinating a complex of signaling proteins including LTCC, PKA, and the β_2_-adrenoceptor at the t-tubule membrane in normal ventricular myocytes (1, 5, 27). Although Cav-3 is important to the constitutive maintenance of *I*_Ca_ by PKA at the t-tubule in normal ventricular myocytes, it does not appear to be required for localizing *I*_Ca_ density at the t-tubule membrane, because the difference in *I*_Ca_ density between t-tubule and surface sarcolemma was maintained after inhibition of PKA (Fig. 6*A*).

#### Regulation of I_Ca_ by PKA in CAL myocytes.

In contrast to sham myocytes, *I*_Ca_ increased in response to β_2_-adrenergic stimulation in both intact and DT CAL myocytes (Fig. 1). Moreover, C3SD had no effect on *I*_Ca_ in CAL myocytes (Fig. 3). However, as in sham myocytes, forskolin increased (Fig. 2), and H-89 decreased (Figs. 4, 5, and 6), *I*_Ca_ at both the surface and t-tubular membranes. The simplest explanation of these data is that the normal Cav-3-dependent localization of β_2_-adrenoceptor signaling at the t-tubules is disrupted in CAL myocytes, so that the β_2_-adrenoceptor is distributed across both the surface and t-tubular membranes and can stimulate adenylyl cyclase/PKA and thus LTCCs at both sites, even without Cav-3 regulation; this is consistent with the redistribution of β_2_-adrenoceptor cAMP signaling in heart failure (27) and demonstrates that Cav-3 is not required for β_2_-adrenoceptor stimulation of adenylyl cyclase/PKA, which are already present at both sites (Fig. 7*B*). Interestingly, in the presence of H-89, *I*_Ca_ density was similar in the t-tubular and surface membranes of CAL myocytes, suggesting that LTCCs are also redistributed in heart failure (6). The mechanisms underlying the redistribution of β_2_-adrenoceptors and LTCCs away from the t-tubules, resulting in a more uniform distribution across the cell membrane, are unclear; presumably, the redistribution of Cav-3 to noncholesterol-rich membranes in heart failure leads to a loss of Cav-3 from the t-tubules and the consequent disruption of Cav-3-dependent complexes containing LTCC/adenylyl cyclase/PKA/β_2_-adrenoceptors (29). Cav-3 likely plays a role in the localization of the β_2_-adrenoceptor to the t-tubule so that the loss of Cav-3 regulation from the t-tubule membrane in heart failure contributes directly to the redistribution of the receptor to the surface sarcolemma and the loss of localization of β_2_-adrenoceptor signaling to the t-tubule in failing myocytes (1, 5, 27, 32). Alternatively, in principle, it is possible that β_2_-adrenoceptors are more uniformly distributed between the t-tubule and surface membranes and that Cav-3 may be responsible for the localization of adenylyl cyclase/PKA signaling to the β_2_-adrenoceptors in the t-tubules. Consistent with either of these proposals, treatment of normal ventricular myocytes with C3SD peptide has been shown to antagonize β_2_-adrenoceptor-mediated increases in *I*_Ca_ (5). Moreover, overexpression of Cav-3 restored the localization of β_2_-adrenoceptor signaling to the t-tubules in failing cells, implying a direct role for Cav-3 in the localization of the receptors and/or receptor signaling to the t-tubules, presumably via binding with the scaffolding domain (32). However, the observation that zinterol stimulates *I*_Ca_ at the surface membrane of CAL myocytes, in which C3SD has no effect on *I*_Ca_, suggests that β_2_-adrenoceptor stimulation can stimulate adenylyl cyclase/PKA even without Cav-3 binding. The role of Cav-3 in the loss of t-tubular localization of β_2_-adrenoceptor signaling in heart failure might be tested in future studies by investigating the effect of C3SD peptide on the response of CAL myocytes to β_2_ stimulation. On the other hand, Cav-3 does not seem to play a direct role in the localization of LTCCs to the t-tubule because *1*) interference of Cav-3 binding to its partners in intact sham myocytes by treatment with C3SD peptide had no effect on *I*_Ca_ in the presence of PKA inhibition (Fig. 4), indicating that PKA activity was required for the Cav-3-dependent regulation of LTCCs, and *2*) PKA was not required for the concentration of *I*_Ca_ at the t-tubule in sham myocytes (Fig. 4). Although Cav-3-dependent regulation of t-tubular *I*_Ca_ by PKA was lost in CAL myocytes, they showed an increased ratio of basal t-tubular *I*_Ca_ density to t-tubule *I*_Ca_ density in the presence of H-89 compared with sham myocytes (CAL: basal −5.9 ± 1.6 pA/pF and H-89 −1.7 ± 0.8 pA/pF; sham: basal −12.9 ± 3.0 pA/pF and H-89 −7.4 ± 1.6 pA/pF), indicating that the contribution of PKA to the maintenance of t-tubular *I*_Ca_ was augmented in heart failure. This is consistent with increased PKA-dependent regulation of basal whole cell *I*_Ca_ in failing human ventricular myocytes (10). Nevertheless, the mechanism for the increased constitutive regulation of t-tubular *I*_Ca_ by PKA in heart failure remains unclear.

#### Functional implications of regulation of I_Ca_ by PKA.

Previous work has shown that *I*_Ca_ occurs predominantly in the t-tubules, in close proximity to RyRs in the SR membrane, allowing efficient coupling between Ca^2+^ entry via *I*_Ca_ and Ca^2+^ release from the SR (21, 28). The present work shows that even in the presence of PKA inhibition, *I*_Ca_ still occurs predominantly in the t-tubules of sham myocytes, suggesting a higher concentration of LTCCs in the t-tubules. The observation that β_2_-adrenoceptor stimulation of *I*_Ca_ is normally localized to the t-tubules is consistent with the importance of this site for the normal regulation of EC coupling and the potential detrimental effects of a whole cell increase of cAMP.

In CAL myocytes, although there was little change in whole cell *I*_Ca_ density, there was redistribution of *I*_Ca_ so that it was more uniformly distributed across the surface and t-tubular membranes. Unless accompanied by parallel redistribution of RyRs, which, to the best of our knowledge, does not occur, the reduced Ca^2+^ entry at the t-tubules will result in less effective coupling of Ca^2+^ entry and release and increased numbers of “orphaned” RyRs, resulting in a smaller, slower Ca^2+^ transient and thus contraction. However, the present work shows that increased local constitutive stimulation of *I*_Ca_ by PKA helps to maintain *I*_Ca_ at the t-tubules, which will help ameliorate these deleterious effects.

It has been proposed that a subpopulation of LTCCs in surface membrane caveolae play a role in cardiac hypertrophy (12, 24). The observation that C3SD has little effect on *I*_Ca_ in DT sham or CAL myocytes suggests that Cav-3 binding has little effect on LTCC function at the cell surface, although it remains possible that downstream effects of *I*_Ca_ are altered.

#### Summary.

The present study shows that Cav-3 plays a vital role in the coordination of PKA-dependent regulation of both basal and β_2_-adrenoceptor stimulation of *I*_Ca_ in myocytes from healthy hearts. The colocalization by Cav-3 is lost in heart failure, and both β_2_-adrenoceptors and LTCCs are redistributed from the t-tubular to surface sarcolemma membranes. The role of Cav-3 in the redistribution in heart failure remains unclear, but the data are consistent with a shift in Cav-3 from cholesterol-rich to noncholesterol-rich membranes (29).

## GRANTS

This work was funded by British Heart Foundation Grants PG/10/91/28644, PG/14/65/31055, and RG/12/10/29802.

## DISCLOSURES

No conflicts of interest, financial or otherwise, are declared by the authors.

## AUTHOR CONTRIBUTIONS

S.M.B. performed experiments; S.M.B. analyzed data; S.M.B., C.H.K., M.B.C., C.H.O., and A.F.J. interpreted results of experiments; S.M.B. prepared figures; S.M.B., C.H.K., C.H.O., and A.F.J. drafted manuscript; S.M.B., C.H.K., M.B.C., C.H.O., and A.F.J. edited and revised manuscript; S.M.B., C.H.K., M.B.C., C.H.O., and A.F.J. approved final version of manuscript; C.H.O. and A.F.J. conceived and designed research.
